# Temporal Quantitative Changes in the Resistant and Susceptible Wheat Leaf Apoplastic Proteome During Infection by Wheat Leaf Rust (*Puccinia triticina*)

**DOI:** 10.3389/fpls.2019.01291

**Published:** 2019-10-23

**Authors:** Christof Rampitsch, Mei Huang, Slavica Djuric-Cignaovic, Xiben Wang, Ursla Fernando

**Affiliations:** Agriculture and Agrifood Canada, Morden, MB, Canada

**Keywords:** leaf rust, yellow rust, apoplastic fluid, effector proteins, proteomics

## Abstract

Wheat leaf rust caused by the pathogenic fungus, *Puccinia triticina*, is a serious threat to bread wheat and durum production in many areas of the world. This plant-pathogen interaction has been studied extensively at the molecular genetics level however, proteomics data are still relatively scarce. The present study investigated temporal changes in the abundance of the apoplastic fluid proteome of resistant and susceptible wheat leaves infected with *P. triticina* race-1, using a label-free LC-MS-based approach. In general, there was very little difference between inoculated and control apoplastic proteomes in either host, until haustoria had become well established in the susceptible host, although the resistant host responds to pathogen challenge sooner. In the earlier samplings (up to 72 h after inoculation) there were just 46 host proteins with significantly changing abundance, and pathogen proteins were detected only rarely and not reproducibly. This is consistent with the biotrophic lifestyle of *P. triticina*, where the invading pathogen initially causes little tissue damage or host cell death, which occur only later during the infection cycle. The majority of the host proteins with altered abundance up to 72 h post-inoculation were pathogen-response-related, including peroxidases, chitinases, β-1-3-endo-glucanases, and other PR proteins. Five days after inoculation with the susceptible apoplasm it was possible to detect 150 *P. triticina* proteins and 117 host proteins which had significantly increased in abundance as well as 33 host proteins which had significantly decreased in abundance. The latter represents potential targets of pathogen effectors and included enzymes which could damage the invader. The pathogen-expressed proteins—seen most abundantly in the incompatible interaction—were mostly uncharacterized proteins however, many of their functions could be inferred through homology-matching with pBLAST. Pathogen proteins also included several candidate effector proteins, some novel, and some which have been reported previously. All MS data have been deposited in the PRIDE archive (www.ebi.ac.uk/pride/archive/) under Project PXD012586.

## Introduction

The fungus *Puccinia triticina* is an obligate parasite that causes leaf rust on wheat. Urediniospores of *P. triticina* germinate on the leaf surface, and germlings enter wheat leaves *via* appressoria which form at open stomata and colonize the apoplastic space with hyphae within 24 h after germination. The life cycle can be completed in approximately 7 days when new urediniospores are formed to initiate a new cycle (Bolton et al., 2008). Wheat leaf rust is a damaging disease, and the *Puccinia* species ranked third in a recent review of the top 10 fungal pathogens of crops (Dean et al., 2012). The rust-host interaction has been studied intensely for many decades and is the subject of regular reviews (e.g., McCallum et al., 2016). Although there is evidence that the plant responds to rust spores very rapidly (Nirmala et al., 2010), the early stage of rust infection is biotrophic, and *P. triticina* does not initially attempt to kill its host. Soon after the apoplastic space has been colonized and if the host immune response can be overcome, the fungus invaginates host cells to form haustoria. These feeding structures are the putative source of most of the pathogen’s effector proteins (Vögele and Mengden, 2003).

While the host is being colonized, it is of course mounting an immune response. In fact, the rust-flax interaction was one of the first plant-pathogen interactions to be studied in detail, leading Flor to formulate the gene-for-gene theory more than 40 years ago. This model has since been enlarged, notably by Jones and Dangl (2006) to include several phases leading up to the *avr-R* gene-for-gene interaction itself, and it continues to evolve (Pritchard and Birch, 2014). Major targets of the host immune system are effector proteins produced by the pathogen, and successful recognition of the pathogen avirulence gene(s) by the host results in a localized hypersensitive reaction which kills the infected host cell and arrests the fungal life cycle. Although resistant plants do present minor leaf symptoms to a varying degree, depending on the *R* gene(s) present, these symptoms are mild, and no sporulation occurs (Bolton et al., 2008).

The apoplastic fluid within wheat leaves is the direct interface between the protagonists and is therefore a potentially rich and interesting source of proteins involved in host defense and pathogen virulence (Martínez-González et al., 2018). In addition, the apoplastic fluid is relatively easy to obtain in sufficient quantity for proteomic analyses as long as great care is taken not to cause too much damage and hence avoid its contamination by intracellular proteins. Since wheat leaves are narrow with parallel veins, the easiest approach to harvesting apoplastic fluid is simply to centrifuge it out. A more complex fluid can be obtained if the leaf is first placed under vacuum and then infiltrated with a buffer of mild ionic strength, as this will release proteins that are weakly bound to the cell wall through non-covalent interactions. However, the increased manipulation and vacuum treatment risks greater damage to cells, especially in seedling leaves and hence greater contamination by intracellular proteins. A further complication is that the apoplastic fluid is likely to contain many diverse proteolytic enzymes and is therefore a hostile environment for keeping proteins intact, thus a short protocol with rapid inactivation of proteases is advantageous (Lohaus et al., 2001).

Aside from pathogenesis-related proteins, frequently described as responding early to infection (reviewed by Rampitsch and Bykova, 2012), researches are also interested in identifying candidate secreted effector proteins (CSEP). The broad function of effector proteins is to manipulate the host’s immune system to benefit fungal growth and survival and in the case of rusts; they have been reported to be secreted mainly from haustoria. The repertoire of effector proteins in the rust fungi is thought to be quite large, with hundreds CSEP available for most species (summarized by Petre et al., 2014; Asahi and Shirasu, 2015). Candidate effector protein lists are often generated using bioinformatics approaches (Sperschneider et al., 2017) using the following criteria: *(i)* possession of a known secretion signal peptide, *(ii)* smaller than 300 amino acids in length, *(iii)* rich in cysteine residues, *(iv)* homology to known effector proteins, and *(v)* specific to the rust genome. Since some of the proteins presented here are likely of haustorial origin, 12 CSEP were highlighted in this study, even though most CSEP are thought to be targeted to the cytosol of host cells (Koeck et al., 2011).

Here, we present a comparative analysis of the proteomes of resistant or susceptible wheat apoplasm colonized by race-1 of *P. triticina* through the first 5 days of infection. We initially compared resistant and susceptible plants separately to uninoculated controls and demonstrated that the host response had a more rapid onset in the resistant apoplasm and that the response was very limited until the final time point measured (5 days after infection). At this point, the susceptible apoplasm yielded 354 *P. triticina* proteins and 150 wheat proteins with increased abundance relative to the resistant apoplasm. These findings are supported by the biotrophic lifestyle of *P. triticina*, in which the fungus feeds off living tissue and only kills host cells much later on during infection. We also compared the resistant apoplasm to the susceptible (both inoculated) which revealed mainly proteins that were more abundant in the susceptible apoplasm—likely a reflection of the higher fungal biomass in this material—but also host proteins which were more abundant in the resistant apoplasm and which could therefore have a role in resistance. From our findings, it appears that the majority of secreted rust proteins come from the haustoria and not from intracellular hyphae, possibly a pathogen strategy to avoid detection by the host immune system for as long as possible to be able to establish a viable infection.

## Materials and Methods

### Biological Material

Bread wheat (*Triticum aestivum* L.) cultivars “Thatcher” (RL6101) and near-isogenic “Thatcher*Lr1*” which bears the *Lr1* leaf rust resistance gene were inoculated with urediniospores of *P. triticina* race-1 (virulence phenotype BBBD) at the one-leaf stage, 8 days after emergence. Spores were mixed with light mineral oil and sprayed onto the leaves using an air powered sprayer. Mock inoculation was performed separately by spraying oil only, and care was taken to keep these control plants physically separated from the experimental plants. After 30 min, plants were transferred to a dew chamber and kept in the dark. The relative humidity was maintained near 100% for 24 h, after which plants were moved to a growth cabinet and grown until harvest, using an 18-h day-length at 24°C and 6 h dark at 18°C. The inoculated leaves were harvested after 24 h, 2 d, 3 d, and 5 d, with a few plants left to grow so that symptoms could be evaluated for each experiment. Three independent biological replicates were performed, starting with new plants.

### Microscopy

Microscopy was performed as described by Wang and colleagues (2013), with minor modifications. Leaves were fixed in 3:1 ethanol:trichloromethane (v/v) containing 0.15% trichloroacetic acid (w/v) for 24 h and washed in 50% (v/v) ethanol. Leaves were then incubated in 0.5 M NaOH at 90°C for 30 min, rinsed with water, and incubated in 0.1 M Tris-HCl (pH 5.8) for 30 min. After staining in 0.1% Uvitex 2B for 5 min, leaves were washed 5 times for 10 min in water and mounted onto microscope slides in 50% (v/v) glycerol. A UV fluorescence microscope was used to observe and photograph the tissue (Zeiss AX10: Zeiss Canada, Toronto ON).

### Preparation of Apoplastic Proteins

Harvested leaves (4.5 g) were cut into 10 cm lengths and rolled into a sheet of Parafilm such that they would fit into the barrel of a 20 ml syringe placed into a 50 ml disposable plastic centrifuge tube containing 50 µl of 4 x protease inhibitor cocktail [Roche Canada, Laval QC]. These were then centrifuged at 1,000*g* for 10 min at 4°C in a swinging bucket rotor. The assembly was then removed and straightened out, followed by repeat centrifugation for 30 min under the same conditions to obtain approximately 100 µl apoplastic fluid. The fluid was then centrifuged at 3,000*g*, 4°C 10 min, and finally at 25,000*g*, 4°C 20 min. Small pellets were discarded at each step. Proteins were precipitated from the final supernatant in 8 volumes of acetone containing 1 mM DTT and 10% (w/v) TCA. After precipitation and washing, proteins were dried under nitrogen and then resuspended in 100 µl of 7 M urea, 2 M thiourea, 4% (w/v) CHAPS, and 20 mM DTT in 20 mM Tris, pH 8. Samples were reduced by incubation at 56°C for 45 min and alkylated with 15 mM iodoacetamide for 30 min at room temperature in the dark. Samples were then centrifuged at 20 min, 13,000*g*, and transferred to dialysis cups (7 kDa cut-off) and dialyzed against three changes of a 25 mM ammonium bicarbonate solution. The protein concentration was measured using a Bradford assay (Bio-Rad Laboratories, Hercules CA, BSA standards), and 100 µg protein was used for trypsin digestion overnight at 37°C [reference]. The digestion was terminated by adding 0.4% (v/v) formic acid; samples were dried under a vacuum and separated into fractions by HPLC.

To compare the protein contents of apoplastic fluids obtained as described above, and obtained by vacuum infiltration as described by Rohringer et al. (1983) and Witzel et al. (2011), samples obtained by each method were separated out by SDS-PAGE and stained with Coomassie Blue. In addition, a total leaf extract was prepared by grinding a single leaf to a fine powder in liquid nitrogen and dissolving the released proteins in SDS-gel loading buffer. Gels were electrophoresed in a Mini Protean II unit (Bio-Rad Laboratories) under conditions recommended by the manufacturer. Bands running at the MW regions of RbcL and RbcS were excised, and their protein identities were determined by bottom-up proteomics/tandem mass spectrometry as described in detail previously (Rampitsch and Bykova, 2009).

### Off-Line Separation of Peptides by RP-HPLC

Once dried, peptides were resuspended in 2 ml mobile phase A1 (10 mM NH_4_OH, pH 10) and fractionated by high pH RP-HPLC as described by (Dwivedi et al., 2008). Briefly, the peptides were separated using a C_18_ column (Thermo Fisher Scientific: Hypersil GOLD 150 x 3 mm, 5 μm particles) attached to an analytical HPLC unit (Ultimate 300: Dionex/Thermo Fisher Scientific, Bremen, Germany). A gradient of mobile phase A1 to 60% mobile phase B1 [10 mM NH_4_OH, pH 10 in 80% (v/v) ACN] was delivered at 0.75 ml.min^−1^. The absorbance of the baseline was monitored at 280 nm, and 30 1 ml fractions were collected throughout. The first 5 ml were discarded, and the remainder was pooled in sets of three by combining every tenth fraction. The pooled fraction 9 was discarded for time points 24 h, 2 d, and 3 d, as these contained an abundant contaminant that produced singly charged ions which suppressed the ionization of peptides; however, this was corrected in the final time point where the full 10 pooled fractions were analyzed. Pooled fractions were dried in a SpeedVac and stored at −20°C until required. Thus, each biological replicate (A, B, and C) contained four interactions, or experimental groups: Oil-Thatcher, Oil-ThatcherLr1 (controls), Rust-Thatcher (susceptible), and Rust-ThatcherLr1 (resistant). Each experimental group contained 9 or 10 samples for a total of 36–40 samples per experimental group. Ultimately, a total of 444 samples were injected into the LC-MS for this study, resulting in 444 .RAW files to be queried (see **Table S1** for details).

### Mass Spectrometry

Pooled fractions were re-dissolved in 20 μl of mobile phase A2 [water containing 2% (v/v) ACN and 0.1 % (v/v) FA]. All three biological replicates were analyzed in a single session, with all control samples injected first to prevent cross-contamination in the column. Tryptic peptides were separated through a C_18_ column (12 cm fused silica column, 75 µm ID, packed with Vydac C_18_, 5 µm beads, 300 Å pores) coupled directly to the mass spectrometer *via* a nanoelectrospray ionization source. An acetonitrile gradient of mobile phase A2 to 40% mobile phase B2 [0.1% (v/v) FA in ACN] was delivered at 300 nl/min over 60 min (Easy nLC 1000: Thermo Fisher Scientific, San Jose CA), with a total program length of 120 min. Mass spectra were acquired in a hybrid quadrupole-Orbitrap mass spectrometer (Q Exactive: Thermo Fisher Scientific, Bremen, Germany). A survey scan acquired over the range m/z 300–2,000 was followed by 12 MS^2^ scans of the most intense ions, with dynamic exclusion set to 15 s.

### Data Analysis

Simultaneous protein identification and label-free quantification (LFQ) was performed using MaxQuant (v1.6) (http://www.biochem.mpg.de/5111795/maxquant). The search parameters were mostly left at default settings; in brief, these were a monoisotopic mass accuracy of ±20 ppm for the first search and ±4.5 ppm for the second, up to two missed cleavages for tryptic peptides, peptide charge up to +7, fixed modification of carbamidomethyl (Cys), and variable modification of oxidation (Met), and acetylation (N-terminus). Raw MS data files were queried against the genomic sequences of *P. triticina* (24,519 sequences) and of *T. aestivum* (145,587 sequences) downloaded from UniProt in August 2017. Four separate searches were submitted, one per time-point (i.e., 24 h, 48 h, 72 h, and 5 d) with four experimental groups per search (Oil-Thatcher, Oil-ThatcherLr1, Rust-Thatcher, and Rust-ThatcherLr1). The search was set up so that analogous fractions among biological replicates were treated as identical, and that peaks from neighboring fractions could be used to generate quantitative data.

The results generated from MaxQuant were analyzed using Perseus (v1.6), which is a companion software to MaxQuant used for statistical analysis (Tyanova et al., 2016a; Tyanova et al., 2016b). The LFQ values generated by MaxQuant were loaded as main columns for statistical analyses. The matrix was then reduced by filtering out proteins only identified by site and proteins with a reversed sequence (decoys). The data were then transformed logarithmically (log_2_), and rows were filtered based on valid values, with at least two required per row, i.e., each identified peptide had to have a valid LFQ value in at least two biological replicates to be included in the final data. In cases where LFQ values were measured only in two of three biological replicates, the missing value was imputed using random numbers generated from the Gaussian distribution of the existing values but down-shifted by 1.8 standard deviations (width set to 0.5 SD) to mimic low abundance protein LFQ values more accurately, as described by Tyanova and colleagues (2016a). Following this, Perseus was used to perform a two-sample Student’s t-test between oil- and rust-inoculated genotypes. A volcano plot was generated with a setting of S_0_ = 1.5 and FDR = 0.05 to determine which proteins were significantly enriched in any experimental group. The shape of the cut-off curve (the volcano) was calculated by Perseus and is described in detail by Rudolph and Cox (2019, and references therein).

The same analysis was performed to compare resistant and susceptible plants inoculated with *P. triticina* race-1, at all four time points.

### Validation of Gene Expression and Fungal Biomass Estimation by Real-Time PCR

To validate the proteomic dataset, the expression of the following wheat genes was examined by RT-PCR: POX3 (W5C8U5_WHEAT) and class III peroxidase, PRX113 (C6ETB3_WHEAT). These genes have been reported previously to be induced at the mRNA level in wheat leaves upon challenge by leaf rust (Kumar et al., 2014) and were increased in abundance in the present proteome dataset. In addition, the expressions of three well-characterized defense-related genes, β 1-3 glucanase (PR2), thaumatin-like protein (PR5), and endochitinase (PR4) (Casassola et al., 2015; Li et al., 2015), were assessed for comparison.

Real time PCR was performed as follows. Total RNA was first isolated from wheat leaves using TRIzol reagent, as described by the manufacturer (Invitrogen, Carlsbad CA). Total RNA was treated with DNase I using the TURBO DNA-Free Kit following instructions by the manufacturer (Ambion/Thermo Fisher Scientific). First strand cDNA was synthesized from 1 μg of total RNA using SuperScript III (Invitrogen) according to the manufacturer’s instructions. Controls lacking reverse transcriptase or lacking template were included. The quantity and purity of RNA were analyzed after each step by gel electrophoresis as well as optical density reading using a NanoDrop Spectrophotometer (Thermo Fisher Scientific). All qPCR analyses were carried out on a CFX96 real-time PCR Thermocycler (Bio-Rad Laboratories). Specific primers were designed using Primer 3.0 as shown in **Table 1**.

**Table 1:** DNA sequences of primers used for real-time PCR.

Target	Gene annotation	Forward sequence	Reverse sequence
POX3	Peroxidase	CTCCTTACGGTCGACATGGT	TGGTGTAGTAGGCGTTGTCG
PRX113	Class III peroxidase	TCAATACGGTCGACATGGTG	GAGTCGTCGTGTCCAGGTTC
Ta-EF	Translation elongation factor α	GGTGATGCTGGCATAGTGAA	GATGACACCAACAGCCACAG
PtRTP1	Rust transferred protein	CGGAAGAATAGCCGGAAAATG	CTTAGACATCTCGATGTCTCG

PCR reactions were performed in a 20 μl and contained SsoFast EvaGreen Supermix (Bio-Rad Laboratories), 2 μl of template, and 250 nM of each primer. Thermal cycling parameters were: 98°C, 2 min; 39 cycles of 95°C, 10 s; and 60°C, 30 s. Three technical replicates were performed on each sample. The specificity of the PCR reaction was determined by melting curve analysis between 65 and 95°C, and the efficiency of each primer was checked using the standard curve method. Primers with slopes between −3.1 and −3.6, and the reaction efficiencies between 90 and 110% were selected for analysis. Transcript levels of wheat genes in *P. triticina*–inoculated leaf were compared to the levels of transcripts of the mock inoculated controls. PCR amplifications were normalized using wheat translation elongation factor 1-α gene (TaEF, GenBank accession no. M90077), and the normalized relative expression was calculated using the delta ∆Ct method (Schmittgen and Livak, 2008). Expression levels of gene transcripts were quantified by the qPCR analysis software version 2.2 (Bio-Rad Laboratories).

To monitor haustoria development, and hence infer biomass accumulation, the single copy gene *PtRTP1* (Rust transferred protein; Song et al., 2011) was assessed using gene-specific primers (**Table 1**). The relative amounts of PCR product of PtRTP1 in infected samples and mock inoculated controls were calculated using gene-specific standard curves with *P. triticina* fungal DNA.

## Results

### Assessing the Quality of the Apoplastic Proteome

**Figure 1:**
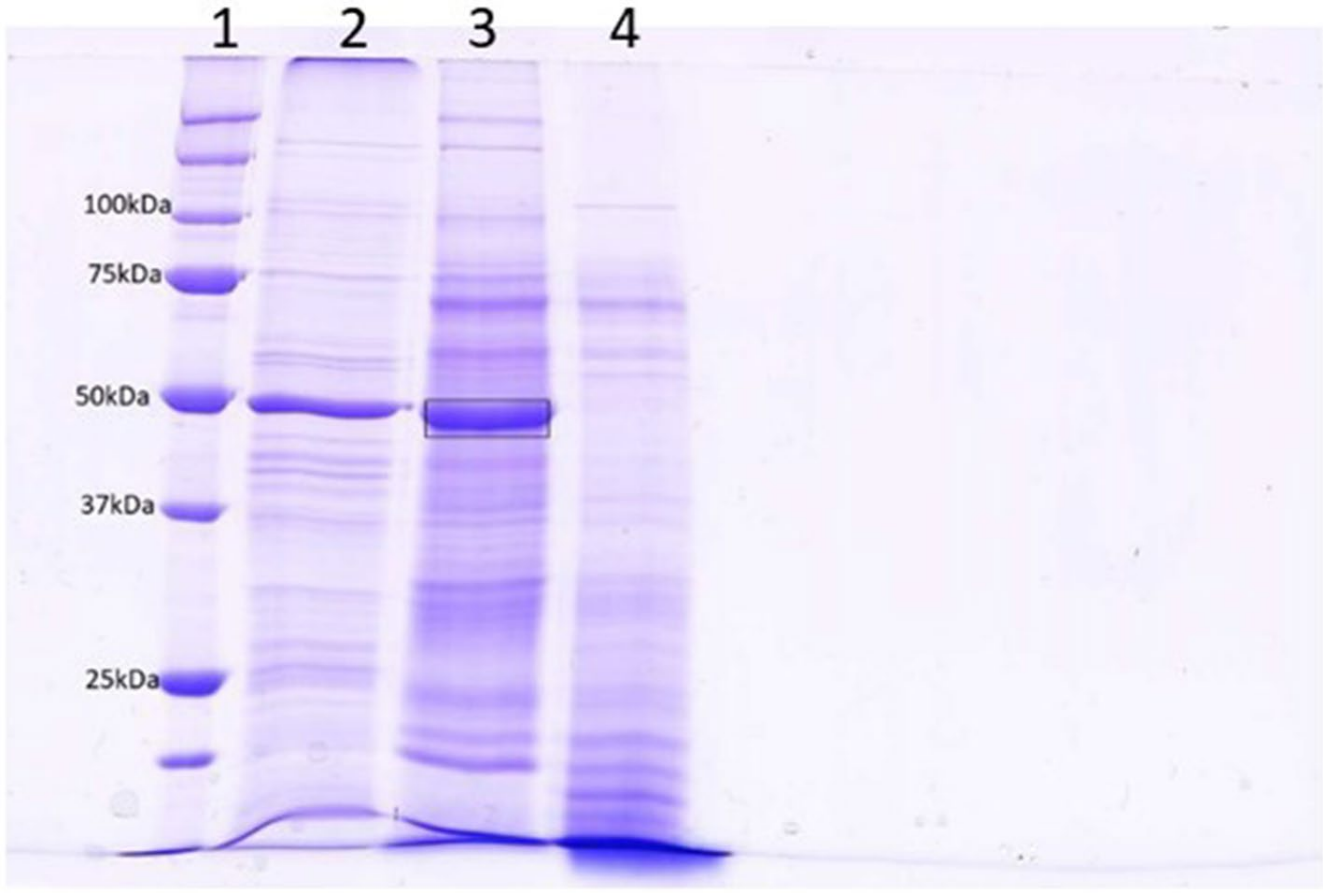
SDS-PAGE analysis of centrifuged (lane 4) and infiltrated (lane 3) leaf apoplastic fluids. Lane 2 contains a total leaf extract and lane 1 is a molecular weight standard. A band from lane 3 (Mr = 50) was excised for analysis by LC-MS (boxed). The results of this analysis is in **Figure S1** and indicates that the most prominent protein in this band was RbcL.

The level of contamination of proteins from damaged cells in the apoplastic fluid is a concern that has been discussed extensively by Lohaus and colleagues (2001). Contamination was initially estimated by assaying malate dehydrogenase activity (Tetlow and Farrar, 1993). The results of these assays were unclear and several other reports had shown that some MDH is in fact present in the apoplastic fluid of some plant species (Li et al., 1989, and discussed by Lohaus et al., 2001). **Table S2** shows LFQ intensities from the wheat cytoplasmic protein actin, chlorophyll protein ribulose bisphosphate carboxylase, and nuclear protein histone H2B, transformed using log_2_. The values indicate that, although these proteins could be detected in all samples, there was no significant enrichment in any experimental treatment; thus, while contamination was present, it was low and even across all samples. **Figure 1** compares the protein content of apoplastic fluid obtained by centrifugation alone to that obtained by prior infiltration with a mild ionic buffer. It can be seen that infiltration releases significant quantities of RuBisCO into the apoplastic fluid, indicative of tissue damage. The band indicated was excised and determined to contain mainly RbcL by mass spectrometry sequencing (**Figure S1**).

### Progress of the Rust Infection

**Figure 2:**
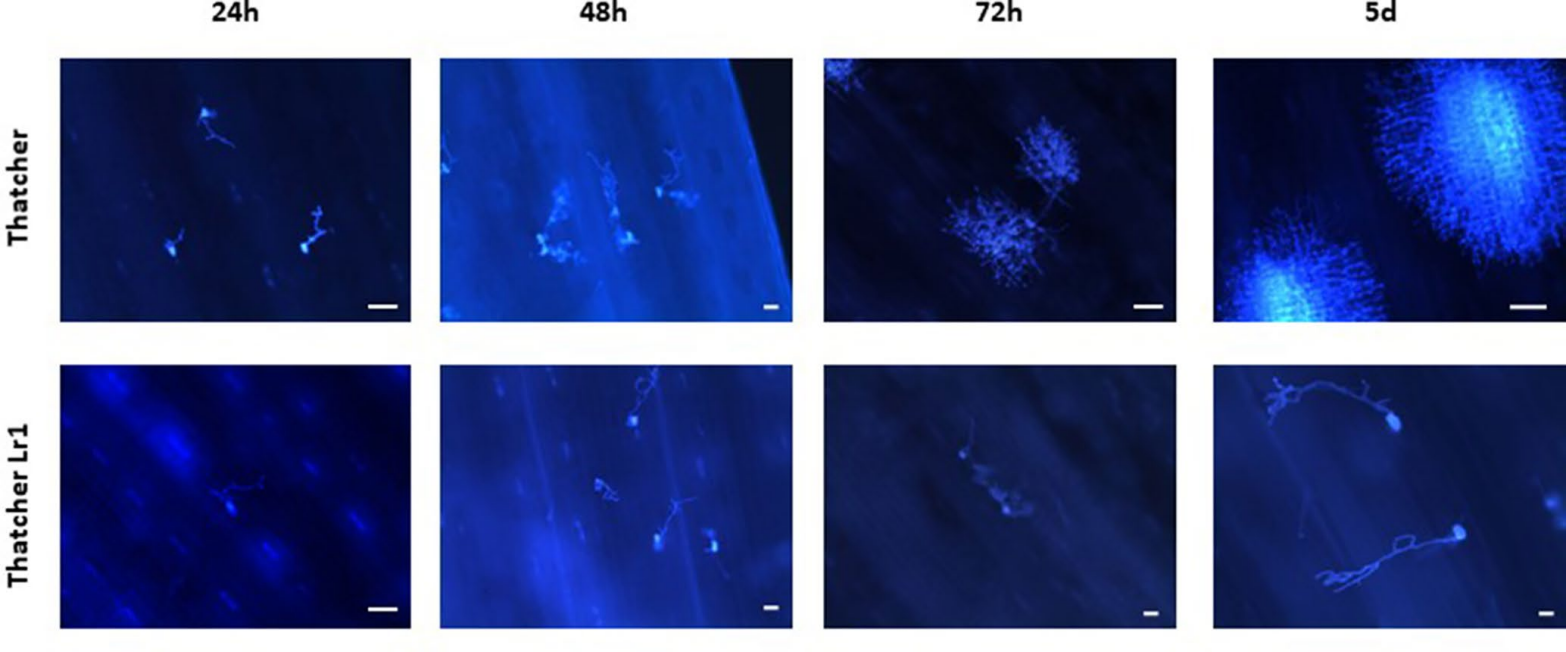
Qualitative view of fungal biomass accumulation shows that more fungal tissue grows during an incompatible interaction on wheat leaves. Micrographs of leaves of the susceptible wheat “Thatcher” (upper) and of the same variety bearing the *Lr1* resistance gene (lower) inoculated with spores of *P. triticina* and harvested at the times shown. Images were made using a UV microscope. The white bar indicates a length of 100 µm.

**Figure 3:**
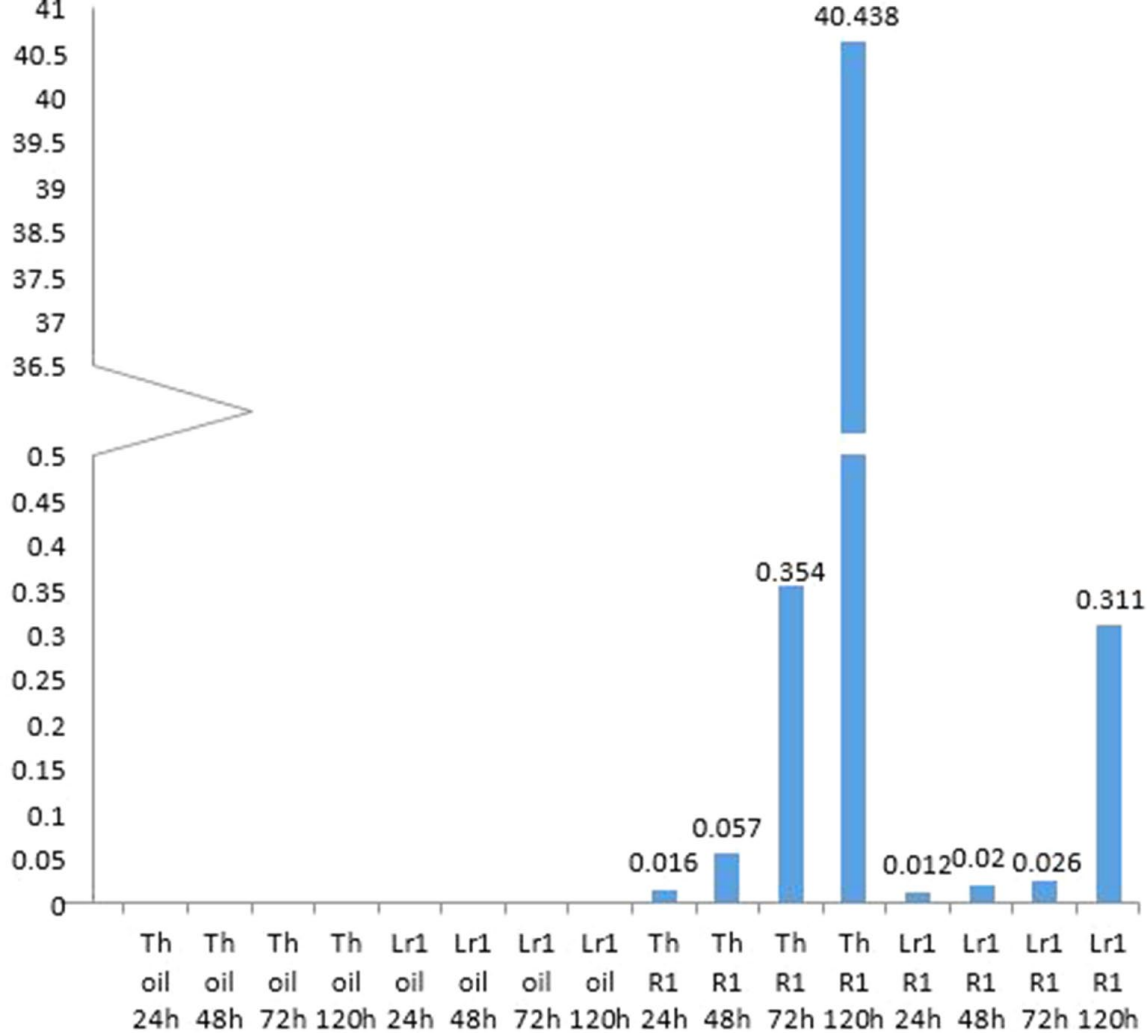
Monitoring haustoria accumulation by real-time PCR shows an exponential accumulation of haustoria-specific RTP1 transcripts in the susceptible cultivar, and that very few haustoria develop if the resistance gene *Lr1* is present.

Micrographs of rusted leaves at all stages in both genotypes investigated here are shown in **Figure 2**. Although the histology of the infection process has been reported in detail previously (Hu and Rijkenberg, 1998; Wang et al., 2013), these images provide a qualitative view of the infection progress. While it is important here to demonstrate the presence of haustoria, this is difficult to do using microscopy of intact tissue because haustoria do not stain well as they are intracellular, and the stain does not penetrate plant cells efficiently. Furthermore, haustoria can be confused with other structures. For this reason, to confirm their presence more accurately, we measured the expression of the *RTP1* gene of *P. triticina* (**Figure 3**), which is specifically expressed in haustoria (Hahn and Mengden, 1997). It can be seen that the overall biomass of the fungal tissue increased greatly by 5 d in the susceptible cultivar “Thatcher,” whereas the *Lr1* resistance gene clearly arrests fungal growth, with very low expression of *RTP1* during the compatible interaction.

### The Apoplastic Proteome During the Biotrophic Phase

**Figure 4:**
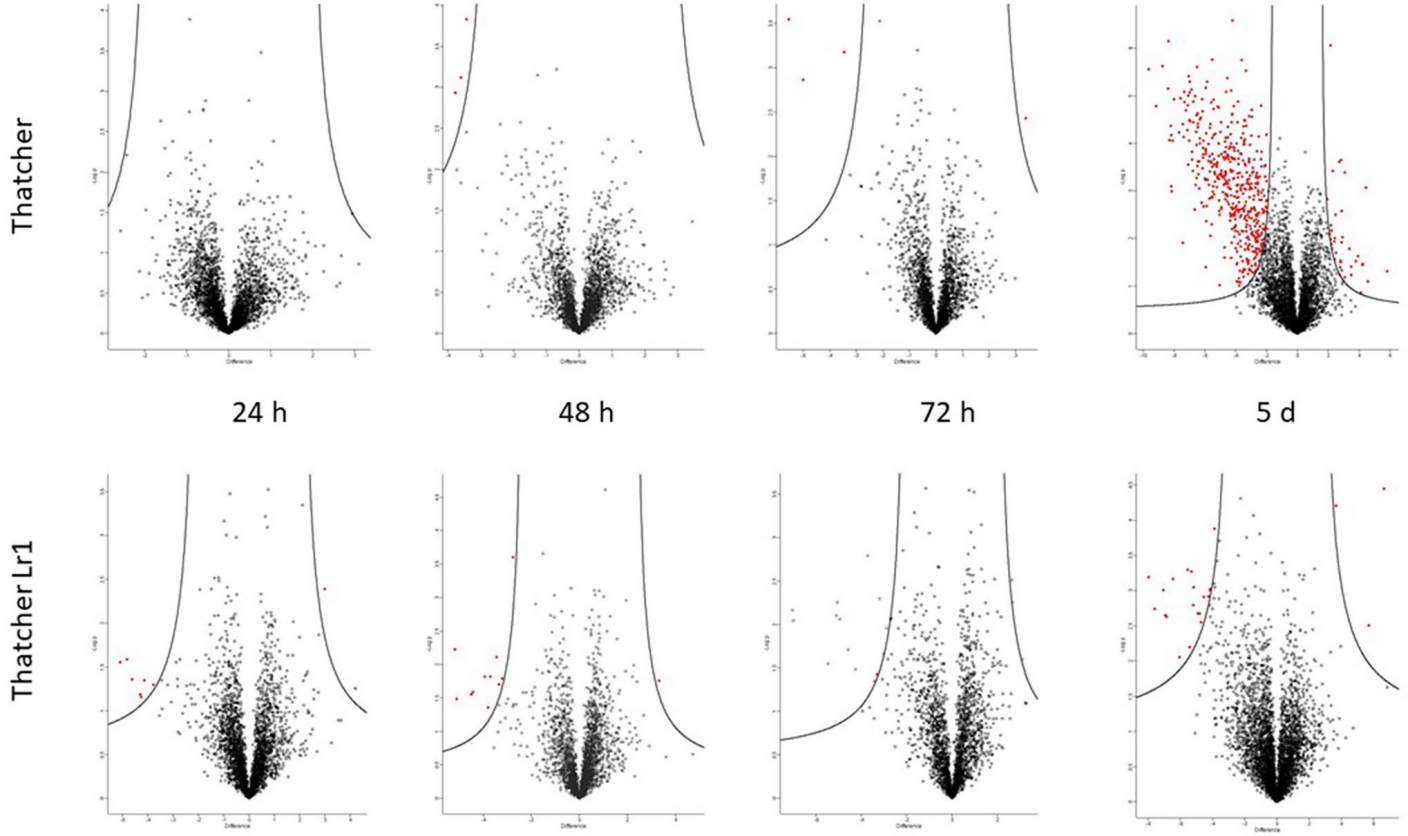
Volcano plots generated by Perseus highlighting proteins whose abundance has changed significantly compared to the uninfected control. Proteins on the left of the volcano decreased in abundance, proteins to the right increased in abundance relative to the control (S_0_ = 1.5 and FDR = 0.05). The putative identities of these proteins are in **Tables 2** and **S3**. It is evident that most of the changes occurred in the susceptible interaction on day 5, at which point haustoria have formed.

**Table S3** lists all proteins whose abundance changed significantly in at least two of three biological replicates in each experimental group. The fold-change in abundance relative to the uninoculated control was calculated based on the precursor ion intensity (“LFQ” or label-LFQ), and data are presented for each sampling time (24 h, 48 h, 72 h, and 5 d post-inoculation). Changes in protein abundance are also shown graphically in volcano plots (**Figure 4**) where proteins falling to the left of the volcano are decreased in abundance relative to the control, and proteins to the right are increased in abundance relative to the control. It can be seen that pathogenesis-related protein 1, peroxidases, and β-1,3-glucanase appear in the rust-resistant apoplasm earlier on, and in greater numbers. However, the response is generally subtle, with fewer than 20 proteins in total responding through increased abundance. There were no *P. triticina* proteins detected in more than a single biological replicate, indicating that *P. triticina* proteins were present in very low abundance (see sect. 3.5). High-confidence proteins from this analysis are in **Table 3**. These are proteins that *(a)* scored above threshold in the MaxQuant analysis, *(b)* were identified by more than one peptide, and *(c)* showed a >2-fold change in abundance. Proteins not meeting all three of these criteria are in **Table S3**.

**Table 2:** Candidate secreted effector proteins from the early harvests. These proteins all possess a known secretion signal (SignalP; see section *Puccinia triticina* proteins), are rich in cysteine, are shorter than 300 amino acids in length, and have no homology to known proteins.

Gene names	Length	Mass	#Cys	%Cys	Harvest
PTTG_25377	98	10335	6	5.4	24, 48, 72
PTTG_10097	106	11146	7	5.8	72
PTTG_11646	118	12453	6	4.6	72
PTTG_11690	118	13126	7	5.3	72
PTTG_27844	127	13879	6	4.3	72
PTTG_12725	127	13919	6	4.3	24, 48, 72
PTTG_10691	134	14522	7	4.7	72
PTTG_12601	139	14702	6	4.0	72
PTTG_12335	160	17769	8	4.5	72
PTTG_01156	183	20035	12	5.8	72
PTTG_09156	254	27473	14	5.0	72
PTTG_06324	296	29956	10	3.2	72

### The Apoplastic Proteome After Haustoria Formation

The volcano plot (**Figure 4**) indicates that there was a substantial increase in proteins (354 *P. triticina* proteins and 150 wheat) with significantly altered abundance 5 days after infection in the susceptible apoplastic fluid only. In stark contrast, the resistant apoplasm yielded four proteins which measured a significant increase in abundance and one with decreased abundance, all of them from the wheat proteome. This is consistent with the amount of fungal biomass seen (**Figure 2**) and measured (**Figure 3**) which were much lower than in the susceptible background. These changes were measured in at least two of three biological replicates and were statistically significant (**Table S3**). These proteins included pathogenesis-related proteins such as chitinases, peroxidases, superoxide dismutases, and β-glucanases as seen in earlier harvests, but also intracellular proteins like ribosomal proteins, metabolic enzymes, and proteasome subunits which indicate that cellular damage has occurred.

Of special interest to plant pathologists are CSPEPs among the *P. triticina* proteins. As discussed by Sperschneider and colleagues (2017), these proteins appear to possess common features (size, cysteine content, lack of homology to known proteins, and having a signal sequence) which identify them as candidates, with the caveat that biological confirmation of this role is difficult and only rarely achieved. **Table 2** lists CSEPs found in this study up to 72 h post-inoculation. Only one of these was also reported previously from rust haustoria (Rampitsch et al., 2015). Just two CSEPs were detected in the 24 and 48 h harvests; however, it is unclear whether CSEP abundance is often too low for detection. Later (5 d) CSEPs likely originate from haustoria and were discussed previously (Song et al., 2011; Rampitsch et al., 2015).

### Expression of Peroxidase Genes by RT-PCR

**Figure 5:**
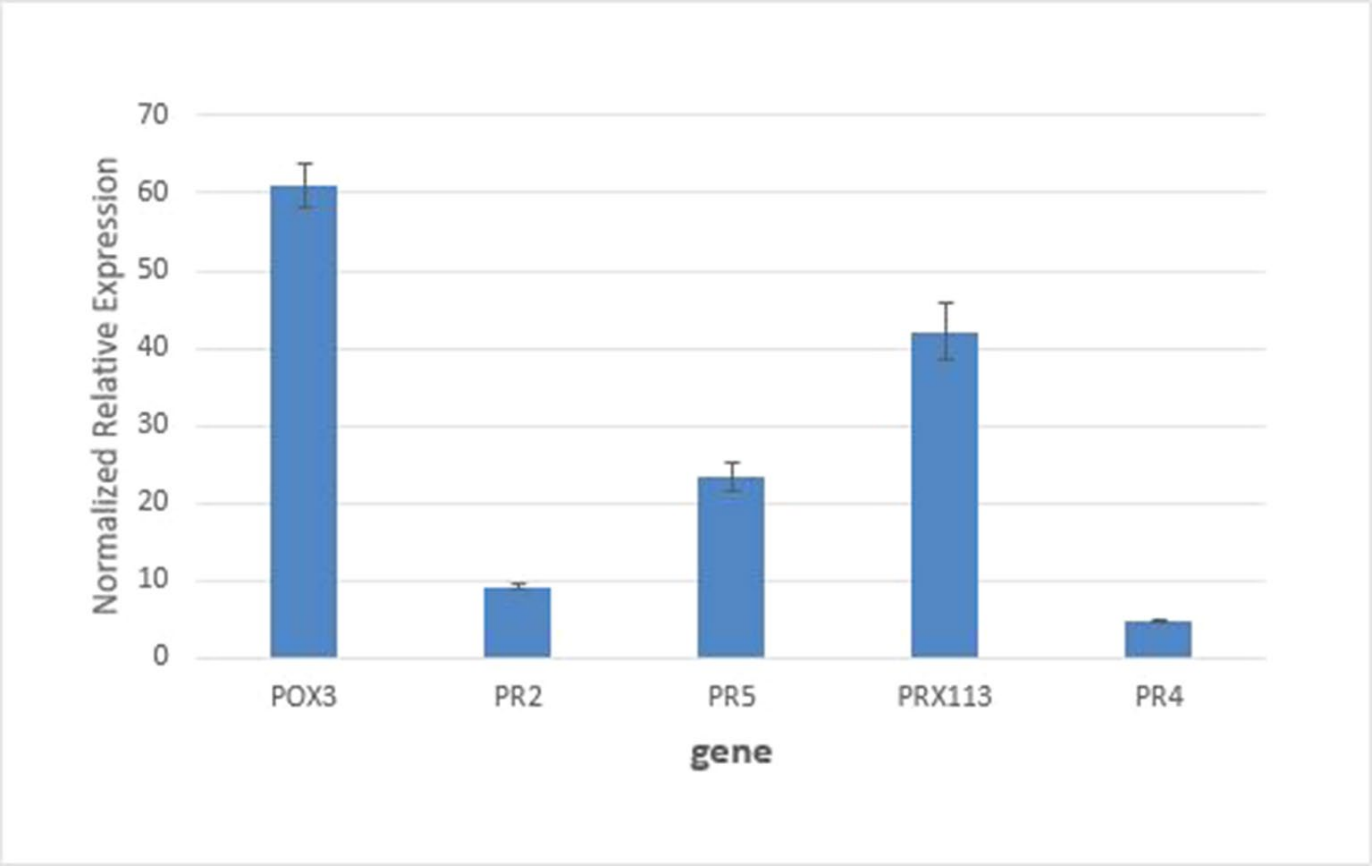
Quantitative RT-PCR analysis of gene expression of POX3 and PRX113 genes coding for proteins W5C8U5_WHEAT and C6ETB3_WHEAT showed an increase of 4.2- and 4.8-fold, respectively, relative to the control. Expression of standard defense related genes PR2, PR5, and PR4 was analyzed for comparison. All pairwise comparisons between genes were statistically significant at P<0.05.

Real-time PCR measurements of mRNA levels of two peroxidase genes encoding W5C8U5_ WHEAT (POX III) and C6ETB3_WHEAT (PRX113) peroxidases in the susceptible host genetic background are shown in **Figure 5**, together with expression levels of commonly used marker genes for biotic stress (PR2, β-1,3-glucanases; PR4, chitin-binding proteins; and PR5, thaumatin-like proteins). It can be see that mRNA levels for the two peroxidases were increased > 4-fold. These results support the findings from the proteomics experiments which saw these proteins increase in abundance by 2.4–3.4-fold in the susceptible apoplasm by 24 h after inoculation.

### Early *P. Triticina* Proteins

Although no proteins from *P. triticina* were significantly altered in abundance prior to 5 d (i.e., in two or more biological replicates), peptides from *P. triticina* proteins were detected in all samples, albeit at low abundance. All *P. triticina* proteins which were detected in only a single biological replicate from the first three harvests are listed in **Table S4**. None of these proteins is within a cluster that also contains homologous wheat proteins and, in many cases, the protein was identified by more than one peptide; however, no statistical tests could be performed.

### Comparing the Inoculated Resistant and Susceptible Apoplastic Proteomes

**Figure 6:**
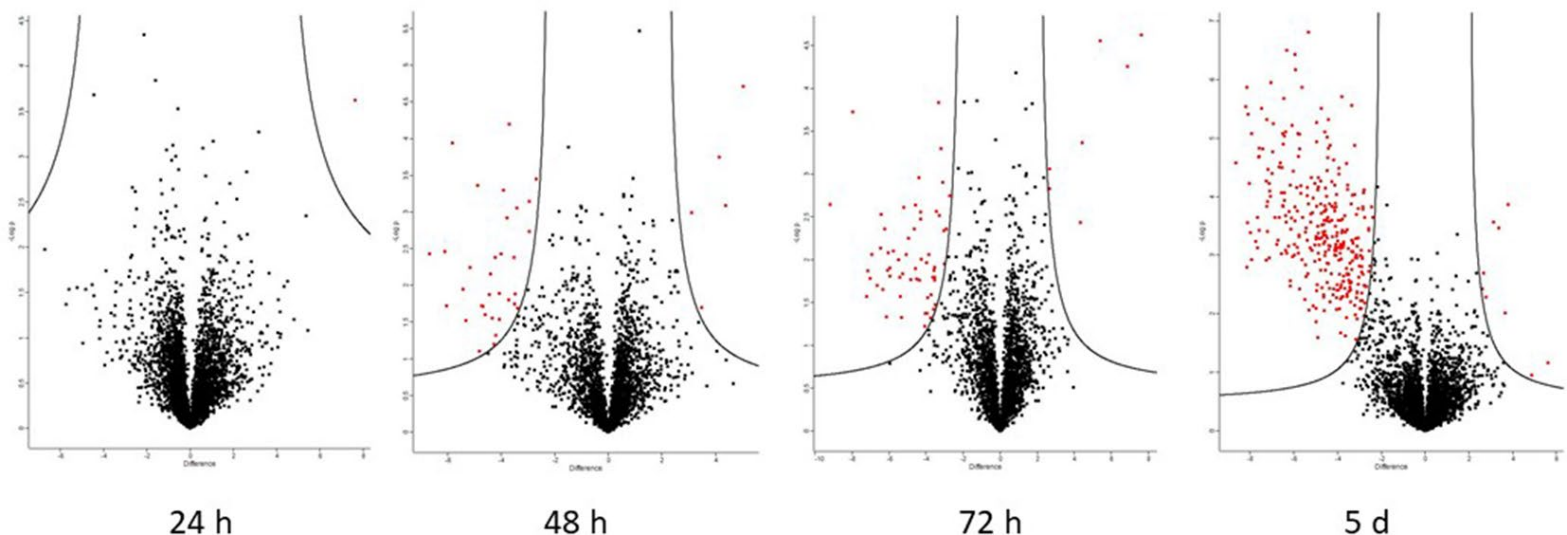
Volcano plots generated by Perseus highlighting proteins whose abundance has changed significantly when comparing resistant and susceptible apoplasm. Proteins on the left of the volcano are significantly more abundant in the resistant apoplasm and *vice versa* (S_0_ = 1.5 and FDR = 0.05). The putative identities of these proteins are in **Tables 3** (high-confidence) and **S5** (complete).

A total of 105 differentially abundant proteins were detected in the first 72 h post-infection. These are proteins whose abundance was altered in the resistant relative to the susceptible apoplastic fluid (**Table S5**). Of these, 83 were identified with high-confidence (as defined by the three criteria in section The Apoplastic Proteome During the Biotrophic Phase). The full data-sets are also represented visually on volcano plots (**Figure 6**) which indicate that the proteome becomes more dynamic as the time after inoculation increases.

Roughly half of all high-confidence proteins changing in abundance in the resistant *vs*. susceptible apoplasm were of unknown function; 6 out of 11 at 48 h and 12 out of 23 at 72 h had neither a known nor putative function (the latter inferred *via* homology searching using BLASTp). Out of the known proteins, a few stress-responsive proteins, such as peroxidase, cyanate dehydratase, and PR proteins such as chitinase and beta-glucanase, were more abundant in the resistant apoplasm. There were three high-confidence proteins more abundant in the susceptible apoplasm: A0A1D5SG39 nucleoside diphosphate kinase, as well as unknown proteins A0A1D5STA6, A0A1D5SF14, W5GDJ5, and A0A1D6BEE8.

## Discussion

### Obtaining High-Quality Apoplastic Fluid

Isolating apoplastic fluid from the leaves of various plants by centrifugation is relatively simple, although care has to be taken that tissue damage is kept to a minimum to prevent contamination by intracellular proteins (Lohaus et al., 2001). Wheat leaves, with their parallel veins and rectangular shape, are well suited to centrifugation. Lohaus and colleagues demonstrated that cytoplasmic contamination of apoplastic washing fluids was negligible if the centrifugal force did not exceed 1,000*g*. Vacuum infiltration with ionic solutions prior to centrifugation has been reported to elute solutes and other substances, including proteins, bound to cell walls through non-covalent interactions (Lohaus et al., 2001; Joosten, 2012). This procedure requires more manipulation, gives more time for proteases to act, and, in our hands, led to the release of RubBisCO (**Figure 1** and **Figure S1**). SDS-PAGE shows that the apoplastic fluids obtained by these methods would yield proteomes of approximately the same complexity; yet, it would not be easy to determine unequivocally which proteins were initially intracellular (i.e., contaminants). We therefore harvested apoplastic fluid using centrifugation only as this would eliminate many contaminant proteins while still yielding a fluid normally inhabited by intracellular hyphae of *P. triticina*. The proteome presented here consequently represents mainly proteins freely inhabiting the apoplastic fluid.

### Progress of the Rust Infection

It is difficult to demonstrate directly which fungal structures are responsible for secreting proteins, because spore germination and fungal growth are not synchronous: different fungal structures form at different times in the host. Haustorial mother cells start to form as soon as 24 h after inoculation; however, at this time, there is still an increase in the numbers of appressoria, a much earlier infection structure (Hu and Rijkenberg, 1998). From the present work, it seems clear that haustoria are the main source of secreted proteins as previously reported by Vögele and Mengden (2003) who measured transcript levels, and by this group (Rampitsch et al., 2015), who examined the proteome of purified rust haustoria. The increase of proteins with significantly altered abundance 5 days post-inoculation can be explained by the increased numbers of haustoria at this time point, especially as this increase was seen only in the incompatible interaction between *P. triticina*-race 1 and Thatcher wheat—the compatible interaction between *P. triticina* race1 and ThatcherLr1 leads to hypersensitive cell death before haustoria can form (Cloutier et al., 2007 and references therein]. However, it is unlikely that all of these proteins originate from haustoria and contaminants resulting from cellular damage by the invading pathogen are also evident. It is important to point out that the fungal biomass seen in the Thatcher-*Lr1* (resistant leaf) 72 h and 5 d panels (**Figure 2**) are likely spores which germinated late, rather than fungal germ tubes which have survived since the inoculation. Non-synchronous germination can effectively cross-contaminate samples from different harvests.

Hu and Rijkenberg (1998) tabulated numbers of specific fungal structures observed through time—however, they did not include haustoria, presumably as these are difficult to identify. They reported that haustorial mother cells, which lead to the formation of haustoria peaked in “Thatcher” wheat—the same cultivar used in the present study—at 96 h (4 d) after inoculation. To confirm the presence of haustoria more accurately, we measured the expression of the *RTP1* gene of *P. triticina* (**Figure 3**). Although expression of this gene is frequently reported to estimate fungal biomass (e.g., Song et al., 2011), it in fact encodes a haustoria specific protein (Hahn and Mengden, 1997) and is more accurately a measure of the presence and relative quantity of haustoria in the sample. It can be seen from the micrographs (**Figure 2**) that the overall biomass of the fungal tissue increased greatly by 5 d in the susceptible cultivar “Thatcher,” whereas the *Lr1* resistance gene arrests fungal biomass accumulation, and this is supported by the real-time PCR measurements, both of which clearly indicated a large increase in biomass in susceptible leaves after 72 h (**Figure 2**).

### The Resistant Apoplasm Secretes Defense-Related Proteins Earlier

Volcano plots were used to identify proteins whose abundance had altered significantly. A volcano plots is a scatter plot of –log_10_ of the *p*-value (Y-axis) and log_2_ of the fold-change on the X-axis. Super-imposed onto this scatter plot are two curves which define the boundaries of proteins whose abundance has been significantly increased relative to the control (on the left side) and significantly decreased relative to the control (on the right side) (Tyanova et al., 2016a). It is also possible to construct three straight lines to define these boundaries, one at y = 1.3 (points above these represent proteins with significant p-values), the other two at x = +1.5 and −1.5 (points outside these represent proteins with a fold-change larger than the user-defined values for the experiment). While this approach yields more significant proteins, many of them are very close to the boundary lines and may be false positives. We therefore used volcano plots (i.e., curves rather than straight lines) to define the significance boundaries, as these produced a more reliable data set.

The most common early-response proteins, with increased abundance in wheat up to 48 h, were enzymes involved in pathogen cell wall degradation and ROS detoxification. Although these proteins were also detected in control plants and in susceptible plants, their abundance increased earlier in the resistant apoplasm. This is consistent with the findings of Kumar and colleagues (2014) who used a wheat Affymetrix gene chip to probe changes in the host transcriptome of transgenic wheat bearing the *Lr1* resistance gene challenged with an incompatible race of *P. triticina*. They reported transcriptional reprofiling as early as 6 h post-inoculation, with most of the up-regulated transcripts encoding defense and stress-related proteins. Studies on *Puccinia striiformis* (Coram et al., 2008) and *Fusarium graminearum* (Bernardo et al., 2007) transcriptomes also revealed early expression of homologues of these genes. It should be stressed that the overall response was low. The reasons for this are that, in spite of a heavy inoculation, the rusted leaf area and fungal growth are relatively low early on. In addition, it is likely that the pathogen is not programmed to secrete many proteins to avoid detection by the host.

Some host proteins showed a decrease in abundance. It has been suggested that these proteins may be targets of pathogen secreted proteases, acting to overcome host defenses (De Jonge et al., 2011). Such changes in protein abundance would not be seen using transcriptome-based approaches since transcript levels would not be decreased as a result of protein degradation. Host proteins with decreased abundance were mostly of unknown function but included also xyloglucan endotransglucosylase, a known apoplastic enzyme responsible for cell wall repair and therefore a legitimate target of the pathogen. More of these proteins were observed in the late harvest and are discussed in section The Susceptible Apoplastic Proteome Changes Dramatically After 5 d.

All our data were compared in two ways: first, each inoculated harvest was compared to its uninoculated control, and second, the resistant and susceptible apoplasms were compared to each other. Since the early harvests, up to 72 h, contain fewer haustoria and are of interest in the early response of wheat to rust, differentially abundant proteins identified with high confidence (defined in Section The Apoplastic Proteome During the Biotrophic Phase) from both comparisons are summarized in **Table 3**, with full data in **Tables S3** and **S5**. All but one of these proteins (A0A1D6D5D4_WHEAT plastocyanin, 48 h) was at higher abundance in the resistant apoplasm. There is a large overlap between proteins of the two analyses, and the majority of identified proteins were pathogenesis-related and not unexpected. The exceptions were a putative somatic embryogenesis receptor kinase 2-like protein (W5DDM8) and putative 60S ribosomal protein L35a-1-like protein (A0A0C4BK99); however, their functions are not clear. Perhaps of interest is the loricrin-like protein (A0A1D6RL92), which in *Phytophthora infestans* is required for oospore formation and plant infection (Guo et al., 2017). In plants, the function of loricin-like protein is to strengthen the cell envelope and thus the defensive barrier (Guo et al., 2017).

**Table 3:** Differentially abundant proteins identified with high confidence (defined in Section The Apoplastic Proteome During the Biotrophic Phase) from both comparisons. All were queried against the nonredundant database of NCBI using the pBLAST algorithm to help identify unknown proteins as far as possible through homology.

Time	Protein IDs	Putative identity	Peptides^a^	Score^b^	pBLAST return
48 h	A0A1D6D5D4	WHEAT plastocyanin	4	317.12	Plastocyanin, chloroplastic
	A0A1D6RL92	WHEAT uncharacterized protein	14	323.31	Loricrin-like
	A0A1D5V896	WHEAT uncharacterized protein	9	127.54	Glucan endo-1,3-beta-glucosidase, acidic isoform-like
	A0A0C4BK99	WHEAT uncharacterized protein	6	43.582	60S ribosomal protein l35a-1-like
	W5C8U5	WHEAT peroxidase	9	263.07	Peroxidase 1-like
	C6ETB3	WHEAT peroxidase	10	313.09	Class III peroxidase
	Q9XEN5	WHEAT beta-1,3-glucanase	12	323.31	Beta-1,3-glucanase precursor
	A0A1D5TI66	WHEAT uncharacterized protein	6	172.8	Ervatamin-C-like
	Q1ERG1	WHEAT endo-beta-1,3-glucanase	11	323.31	Endo-beta-1,3-glucanase
	O82716	WHEAT glucan endo-1,3-beta-D-glucosidase	17	281.7	Glucan endo-1,3-beta-D-glucosidase
	Q94F73	WHEAT pathogenesis-related protein 1	9	323.31	Pathogenesis-related protein PRB1-3
	A0A1D6BJ70	WHEAT uncharacterized protein	12	263.67	Aspartyl protease family protein At5g10770-like
	A0A1D5YUG0	WHEAT uncharacterized protein	4	209.67	Thaumatin-like pathogenesis-related protein 3
	C3UZE5	WHEAT pathogenesis-related protein 1-1	7	293.7	Pathogenisis-related protein 1.1
	A0A1D5TQY9	WHEAT peroxidase	11	323.31	Peroxidase 1
	O82714	WHEAT pathogenesis-related protein 1-3	8	323.31	Pathogenisis-related protein 1.1
	A0A0H4TM98	WHEAT chitinase	8	107.19	Chitinase
	A0A1D5SA13	WHEAT uncharacterized protein	3	323.31	Chitinase 8-like
	Q41584	WHEAT thaumatin-like protein	3	323.31	Thaumatin-like protein
72 h	A0A1D5SU87	WHEAT uncharacterized protein	22	323.31	Chitinase 8-like
	Q41584	WHEAT thaumatin-like protein	3	323.31	Thaumatin-like protein
	F8S6U9	WHEAT pathogenesis-related protein 1-19	5	105.85	Pathogenesis-related protein 1-19
	W5C8U5	WHEAT peroxidase	9	323.31	Peroxidase 1-like
	A0A1D5V896	WHEAT uncharacterized protein	10	323.31	Glucan endo-1,3-beta-glucosidase, acidic isoform-like
	D8L9Q2	WHEAT glucan endo-1,3-beta-glucosidase GII	14	323.31	Glucan endo-1,3-beta-glucosidase GII precursor
	Q9XEN5	WHEAT beta-1,3-glucanase	12	323.31	Beta-1,3-glucanase precursor
	Q4JK90	WHEAT beta-1,3-glucanase	18	323.31	Beta-1,3-glucanase
	O82716	WHEAT glucan endo-1,3-beta-D-glucosidase	18	323.31	Glucan endo-1,3-beta-D-glucosidase
	C6ETB3	WHEAT peroxidase	11	323.31	Class III peroxidase
	A0A1D5UH99	WHEAT uncharacterized protein	10	152.63	Chitinase 5-like
	O82714	WHEAT pathogenesis-related protein 1-3	11	97.119	Pathogenisis-related protein 1.1
	A0A1D5W1T2	WHEAT uncharacterized protein	13	323.31	Glucan endo-1,3-beta-glucosidase GIII-like
	Q9SQG3	WHEAT PR-4 (fragment)	6	211	PR-4, partial
	Q1ERG2	WHEAT endo-beta-1,3-glucanase	9	200.56	Endo-beta-1,3-glucanase
	A0A1D5TQY9	WHEAT peroxidase	11	323.31	Peroxidase 1
	A0A1D6BV27	WHEAT uncharacterized protein	13	323.31	PR17d precursor
	A0A1D5SA13	WHEAT uncharacterized protein	3	322.23	Chitinase 8-like
	Q43212	WHEAT peroxidase	10	323.31	Peroxidase
	W5DDM8	WHEAT uncharacterized protein	8	298.38	Somatic embryogenesis receptor kinase 2-like
	C3UZE5	WHEAT pathogenesis-related protein 1-1	12	323.31	Pathogenisis-related protein 1.1
	A0A1D5TI66	WHEAT uncharacterized protein	7	294.77	Ervatamin-C-like
	A0A1D5V895	WHEAT uncharacterized protein	11	225.1	Glucan endo-1,3-beta-glucosidase GII precursor, putative, expressed
	A0A1D5YUG0	WHEAT uncharacterized protein	4	238.3	Thaumatin-like pathogenesis-related protein 3
	A0A1D6BC87	WHEAT uncharacterized protein	10	117.77	
	A0A077S0N0	WHEAT uncharacterized protein	12	323.31	Glucan endo-1,3-beta-glucosidase, acidic isoform-like
	Q1ERG1	WHEAT endo-beta-1,3-glucanase	11	323.31	Endo-beta-1,3-glucanase
	A0A1D6BJ70	WHEAT uncharacterized protein	12	323.31	Aspartyl protease family protein At5g10770-like
	A0A1D5X5E7	WHEAT uncharacterized protein	9	323.31	Thaumatin-like protein TLP8
	A0A1D5WHM8	WHEAT uncharacterized protein	12	148.36	Glucan endo-1,3-beta-glucosidase GIII-like
	A0A1D5V4E2	WHEAT uncharacterized protein	16	43.778	Glucan endo-1,3-beta-glucosidase GII-like

a The number of peptides observed.

b Andromeda score, defined by Tyanova et al. (2016a). All of the proteins reported here scored above the threshold.

### The Susceptible Apoplastic Proteome Changes Dramatically After 5 D.

*P. triticina* in as obligate biotroph that infects and derives its nutrients from living tissue and does not produce toxins to kill its host. Nutrients are acquired using specialized feeding structures called haustoria, which form inside leaf mesophyll cells from haustorial mother cells. Haustoria are metabolically very active, providing the fungus with nutrients, energy, and also secreting effector proteins to continue to overcome the host’s immune system (Vögele and Mengden, 2003; Rampitsch et al., 2015). There are two main reasons for the increase in apoplastic proteome complexity in the 5 d harvest of susceptible leaves. Firstly, the formation of haustoria would lead to an increase in secreted proteins and, secondly, increasing cellular damage during this later stage leads to more intracellular proteins being identified. This makes it impossible to interpret changes in the natural apoplastic fluid as the contaminant proteins are not secreted and not intended for the apoplastic fluid. It is likely however that the apoplastic space contains CSEPs, and these are discussed in section *Puccinia triticina* proteins.

The number of host proteins with a lower abundance at the 5 d harvest also increased. In addition to proteins of unknown function, these also included peroxidases, superoxide dismutase, dirigent protein, β-glucosidase, pectin esterase, and lipid transfer proteins, indicating that this approach likely is revealing pathogen effector targets. Fungal effectors are known to target host defense proteins; however, only a few have been confirmed (e.g. *Cladosporum fulvum* AVR2 (Shabab et al., 2014]) and most remain unknown (Giraldo and Valent, 2013). Plant defense proteins, such as those identified here, are obvious targets. Identifying the pathogen effector proteins themselves is of course more challenging.

### *Puccinia triticina* Proteins

Within the first 48 h of infection, only a few (up to 32) *P. triticina* proteins were detected in the apoplastic fluid; however, *P. triticina* proteins were also seen in the uninoculated controls, in spite of several precautions being taken to prevent cross-contamination of controls with experimental material, both during growth and analysis of material. These precautions included growing control and experimental plants separately, isolating control apoplastic fluid first, and running all control samples through chromatography columns and through the LC-MS prior to the experimental material. Setting aside all *P. triticina* proteins seen in controls, it is unclear which of the remaining proteins are simply contaminants. In the 24 h sampling, 8 of the top 10 most intense proteins were found in both the control and experimental samples. In the 48 h sample, this number was 7 out of the top 10. This suggests that intracellular hyphae are not secreting many proteins, at least not in large quantities, and this is perhaps consistent with the biotrophic lifestyle which rusts employ: in the early stages of infection, hyphae invade the apoplastic space where evasion or suppression of the plant immune system is a key feature; effector protein secretion by haustoria rather than hyphae would support this (Koeck et al., 2011). More than half of the *P. triticina* proteins up to 48 h were of unknown function (although some putative functions could be inferred from homology searches using pBLAST), the remainder being mostly metabolic enzymes and other intracellular proteins, indicating that there was likely some damage to hyphae during centrifugation. Approximately, 12% of these had a known secretion signal as determined by the SignalP algorithm, and of these, none was detected in the haustoria proteome reported previously (Rampitsch et al., 2015). In view of the risk for claiming functions based on false positive results, no further inferences will be made for these early rust proteins; however, they appear in **Table S4**. Finding *P. triticina* proteins in uninoculated plants is a common occurrence even if precautions are taken; however, it indicates that the extraction and analysis system being used are very sensitive. Furthermore, one should be cautious using the SignalP algorithm, since any such algorithm is necessarily trained on known signal sequences.

The situation changed at 72 h, with 173 *P. triticina* proteins detected in the “Thatcher” apolastic fluid and 26 in the “ThatcherLr1” apoplastic fluid. Again, all of these proteins were seen in only one of three biological replicates, so no statistical tests could be performed. It is still unclear whether these are merely contaminants from broken hyphae; however, by 72 h, only 1 of the top 10 most intense proteins was present also in the controls. Furthermore, some of these proteins, such as PTTG_25377, are classified CSEP, using current predictions (Sperschneider et al., 2017): PTTG_25377 contains a known secretion signal, is relatively small with a mass of 10 kD, and contains six cysteine residues (5.4% of its composition). This protein, along with one other CSEP (PTTG_12725) were detected as early as 24 h after inoculation in the resistant background only—however, detection was not reproducible, and this observation does not preclude the presence of these proteins in the susceptible apoplasm at a low level. In the 48 h harvest, PTTG_12725 was detected at low levels in both apoplasm types. **Table 2** contains a complete list of CSEPs seen in this study up to 72 h post-inoculation. Validating an effector protein function for these candidates is difficult because no robust assays exist. Furthermore, neither the host nor pathogen can be easily transformed, and there is a large number of candidate effectors, some of which may be functioning redundantly. It remains unclear whether these CSEPs originate from haustoria or from other earlier structures since these structures do not form synchronously. Since CSEPs from the 5 d harvest were almost certainly haustoria-derived, these were not included in **Table 2**, as they have been published previously from monoclonal antibody purified haustoria from the same race of leaf rust (Rampitsch et al., 2015).

The number of CSEPs found in this study increased with time, with two being observed throughout from 24 to 72 h (**Table 2**). Targets of the CSEPs are not generally known, and even their validation as effectors is problematic in rusts. In the biotroph, *Blumeria graminis*, a few such targets have been identified—for example, Pennington and colleagues (2016) used a yeast 2 hybrid assay to demonstrate that BEC1054 interacts with glutathione-S-transferase, a malate dehydrogenase, and a pathogen-related-5 protein isoform *in vitro*. More recently, Sabelleck and Panstruga (2018) showed that the CSEP ROPIP1 interacts with the barley protein RACB. In the rusts, avirulent spores have been shown to activate a receptor-like kinase (Rpg1) in *P. graminis* (Nirmala et al., 2011). They demonstrated that this interaction occurred prior to haustoria formation, indicating that effector proteins can originate from tissue other than haustoria. Any proteins decreasing in abundance identified in this study could be targets of effector protein action; however, direct interactions were not demonstrated here.

At 5 d, post-inoculation there were still only 3 *P. triticina* proteins detected in the resistant cultivar, ThatcherLr1, in 2/3 biological replicates. This again reflects the low biomass of rust in this wheat background. The susceptible wheat however had 405 *P. triticina* proteins in the apoplastic fluid. Of these, 354 were seen in more than one biological replicate and can be described as being significantly increased in abundance. These included proteins from many ontological groups and contamination from damaged hyphae, and other fungal structures cannot be excluded.

## Conclusion

This study presents a detailed investigation of changes to the host apoplastic proteome of resistant and susceptible wheat leaves infected with *P. triticina* race 1. We used a simple extraction process to recover an apoplastic fluid that was largely free of contaminants resulting from damaged tissue, and an extensive sample fractionation scheme and a high-resolution mass spectrometer to ensure maximum sensitivity. The results indicated that the resistant wheat apoplasm responded to the invading pathogen sooner by producing defense enzymes and other proteins. It was apparent that the pathogen did not secrete much protein during the early phases of infection, perhaps as a strategy to evade the host immune system. Once haustoria had formed, and likely in the face of extensive tissue damage at the later stages of infection, the apoplastic proteome changed dramatically. In the resistant leaf, where pathogen growth is arrested prior to the formation of haustoria, few changes to the apoplastic proteome were detected as late as 5 d after inoculation. This study also suggests that the pathogen is targeting host defense proteins using its effector arsenal, but that the bulk of the pathogen effector proteins is likely secreted by haustoria rather than earlier structures. The challenge of confirming effector protein function *in vivo* remains.

## Data Availability Statement

The datasets have been deposited in the PRIDE archive (www.ebi.ac.uk/pride/archive/) under Project PXD012586.

## Author Contributions

CR initiated the research, planned the experiments, analyzed the data and wrote the manuscript. MH Performed peptide separations, mass spectrometry and assisted with data analysis. SD-C prepared all of the biological material. XW helped plan the experiment, helped perform the microscopy and identified fungal structures. UF performed the real-time PCR measurements. All authors critically read the manuscript and provided constructive criticism.

## Funding

This work was funded by an internal grant from Agriculture and Agrifood Canada to CR and XW.

## Conflict of Interest

The authors declare that the research was conducted in the absence of any commercial or financial relationships that could be construed as a potential conflict of interest.

## Supplementary Material

Table S1 Samples from HPLC for LC-MS.

Table S2 LFQ intensities for intracellular proteins.

Table S3 Perseus data showing all proteins with significantly altered abundance relative to control at all time-points.

Table S4 Low abundance *Puccinia triticina* proteins.

Table S5 Perseus data showing all proteins with significantly altered abundance.

Figure S1 Mascot return for the gel slice cut from Lane 3, **Figure 1**. Proteins in this gel slice were digested in-gel with trypsin and the peptides extracted. These were analyzed by LC-MS and the resulting RAW file was used to query the nonredundant protein database held at NCBI, limited to green plants using Mascot 2.4. The return shows a 50% coverage of RbcL as the highest-scoring at the protein level with a total score of 15860, indicating the presence of abundant RbcL in this gel slice.
