# Supplementary figures and images for: Temporal Quantitative Changes in the Resistant and Susceptible Wheat Leaf Apoplastic Proteome During Infection by Wheat Leaf Rust (Puccinia triticina)
Source: Front Plant Sci. 2019 Oct 23;10:1291. doi: 10.3389/fpls.2019.01291 (PMC6819374; doi:10.3389/fpls.2019.01291)

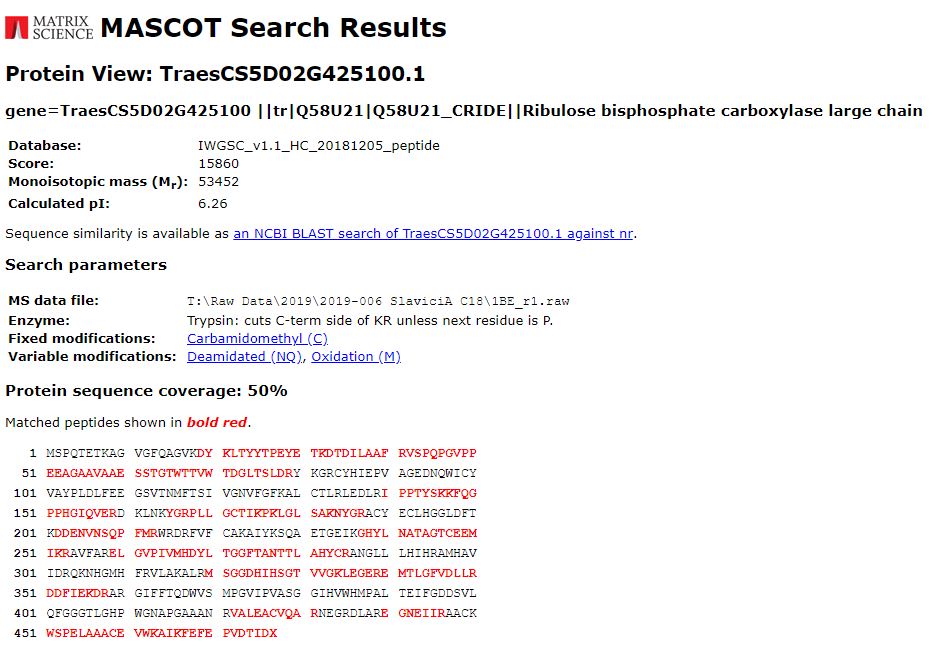

Supplement: Figure S1 — Mascot return for the gel slice cut from Lane 3, Figure 1 . Proteins in this gel slice were digested in-gel with trypsin and the peptides extracted. These were analyzed by LC-MS and the resulting RAW file was used to query the nonredundant protein database held at NCBI, limited to green plants using Mascot 2.4. The return shows a 50% coverage of RbcL as the highest-scoring at the protein level with a total score of 15860, indicating the presence of abundant RbcL in this gel slice. [file Image_1.jpeg]
